# Mortality of Liverpool

**Published:** 1864-03

**Authors:** 


Mortality of Liverpool.—The annual report of the medical officers of this borough states that during the last year the deaths registered in the borough amounted to 15,266—a number greater than in any year since 1849, and 524 above the corrected average of the last decenniad.

